# Broad ligament defect internal hernia: a rare cause of bowel obstruction in pregnancy

**DOI:** 10.1093/jscr/rjag335

**Published:** 2026-04-30

**Authors:** Bader K AlQusaibi, Omar A Bamalan, Nuaman A Danawar, Wael A Zaki

**Affiliations:** General Surgery Department, Security Forces Hospital, PO Box 9003, King Fahad Suburb, 95th Street, Dammam 31413, Saudi Arabia; General Surgery Department, Security Forces Hospital, PO Box 9003, King Fahad Suburb, 95th Street, Dammam 31413, Saudi Arabia; General Surgery Department, Security Forces Hospital, PO Box 9003, King Fahad Suburb, 95th Street, Dammam 31413, Saudi Arabia; General Surgery Department, Security Forces Hospital, PO Box 9003, King Fahad Suburb, 95th Street, Dammam 31413, Saudi Arabia

**Keywords:** pregnancy, bowel obstruction, internal hernia, laproscopy in pregnancy

## Abstract

Internal herniation through the broad ligament is an exceptionally rare cause of small bowel obstruction (SBO), particularly in pregnant patients, and is often misdiagnosed due to non-specific clinical manifestations. This is a 35-year-old woman at 9 weeks’ gestation, medically free, presented to the emergency department with abdominal pain associated with persistent vomiting. Despite conservative management, the patient was deteriorating. Therefore, a patient-shared decision for diagnostic laparoscopy was performed. Intraoperatively, a reduced viable loop of distal ileum was herniated through a defect in the right broad ligament. The postoperative course was uneventful. The persistence of clinical deterioration despite conservative therapy should prompt urgent surgical evaluation, and when indicated, laparoscopy is considered safe in pregnancy and provides diagnostic and definitive management. This case emphasizes that signs of SBO should have a low threshold for early multidisciplinary evaluation, and timely diagnostic laparoscopy is crucial to prevent delayed diagnosis and adverse maternal-fetal outcomes.

## Introduction

Broad-ligament internal hernia represents only a small fraction of internal hernias (commonly cited around 4%–7%) and is an uncommon etiology of small-bowel obstruction, with presentations often nonspecific and initial imaging frequently non-diagnostic (2,3). In early pregnancy when physiologic leukocytosis, overlapping gastrointestinal symptoms, and cautious use of imaging modalities can blur the clinical picture delayed recognition may lead to closed-loop obstruction, bowel ischemia, and increased maternal–fetal morbidity (4,5). Recent case-based surgical literature emphasizes that broad-ligament defects may present without classical peritoneal signs and can closely mimic other surgical or obstetric conditions, making repeat clinical assessment and escalation of care essential when symptoms persist or evolve (6). Contemporary guidelines supports the use of laparoscopy during pregnancy when clinically indicated, with pregnancy-adapted techniques (e.g. modified port placement). This case is educational because it illustrates a rare, high-risk surgical cause of persistent vomiting and abdominal pain in early pregnancy that can be easily misattributed to hyperemesis gravidarum (1). In addition, it highlights a practical, guideline-consistent approach involving early multidisciplinary collaboration and timely diagnostic laparoscopy to both diagnose and treat a rare internal hernia while preserving favorable maternal and fetal outcomes (7).

## Case presentation

The patient is a medically free, 35-year-old pregnant woman, at 9 weeks of gestation, of Middle Eastern ethnicity, presented to the Emergency Department with a recurring, primary complaint of persistent vomiting (approximately seven episodes over two days), intolerance to oral fluids, and upper abdominal pain (predominantly epigastric and para-umbilical in location), over the past 2 days, and denied other symptoms (e.g. fever, urinary symptoms, weight loss). Her past surgical history was significant for a laparoscopic sleeve gastrectomy and two cesarean sections, performed approximately six years prior, with no reported postoperative complications. Upon further evaluation, there was no significant family history (e.g. for malignancy or autoimmune disorders), no substance use disorder or any psychosocial dysfunctions.

Clinically, the patient was hemodynamically stable and afebrile, with vital signs within acceptable limits for early pregnancy. She appeared comfortable at rest, was alert, oriented, and in no acute distress. There were no signs of dehydration, pallor, or respiratory compromise, with an unremarkable cardiovascular and respiratory examinations. However, the abdominal examination revealed localized tenderness over the epigastric and supra-umbilical regions, without guarding, rigidity, or rebound tenderness.

Laboratory investigations demonstrated leukocytosis (white blood cell count up to 19.5 × 10^9^/L) with mild anemia, along with electrolyte disturbances (hyponatremia and hypokalemia), likely related to persistent vomiting. The Inflammatory markers were mildly elevated initially, with a subsequent rise in C-reactive protein during admission despite other laboratory investigations being unremarkable (e.g. liver function tests, urinalysis).

**Figure 1:**
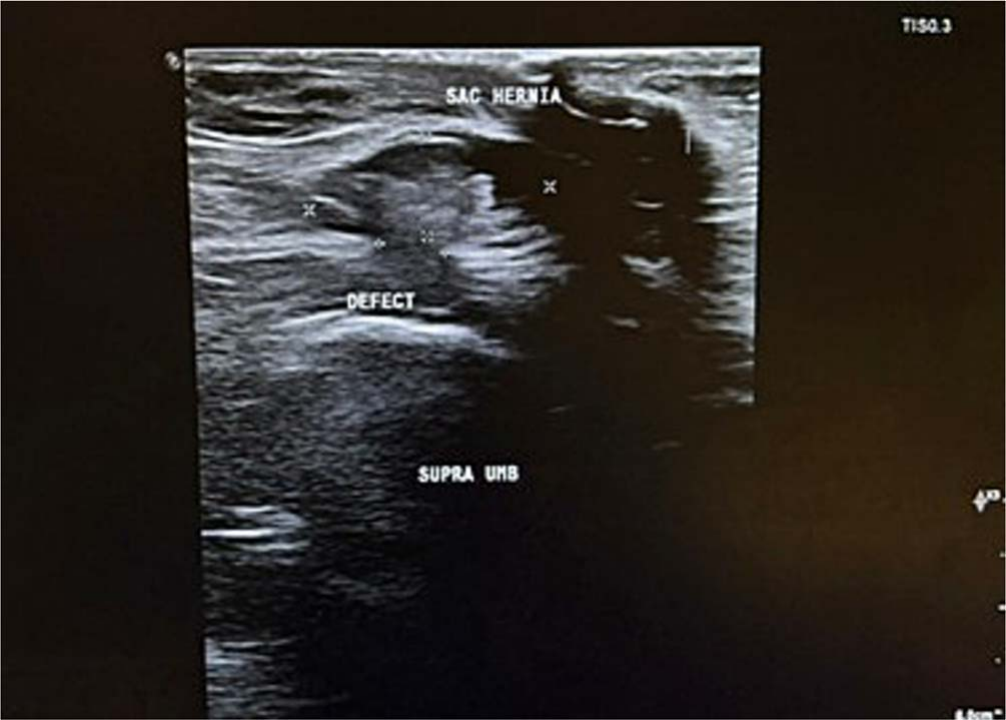
US pic showing interval small bowel dilatation with to-and-fro peristalsis, moderate free fluid in the pelvis and upper abdominal recesses, and a small supra-umbilical abdominal wall defect with a hernia sac containing fluid and a possible bowel loop.

Radiologically, an initial transabdominal abdominal ultrasound (US) was done, showcasing no evidence of an acute inflammatory process (e.g. acute cholecystitis), while the repeated US after the inflammatory markers rise showed multiple findings (Fig. 1).

**Figure 2:**
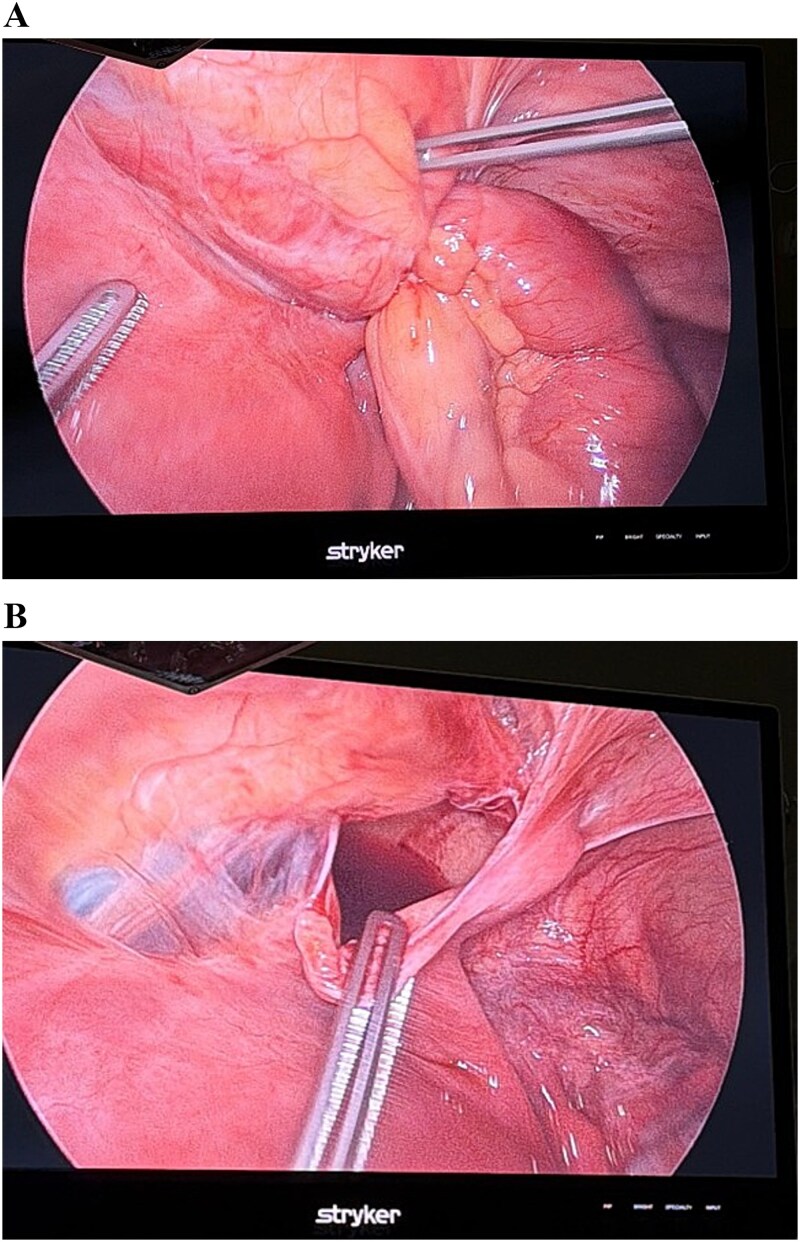
A and B showing a reduced viable loop of distal ileum was found herniated through a defect in the right broad ligament.

Given the persistent symptoms, laboratory markers and equivocal radiological findings, a patient-shared decision to proceed with a diagnostic laparoscopy was performed. Intraoperatively, a reduced viable loop of distal ileum was found herniated through a defect in the right broad ligament, with associated small bowel dilatation and free fluid (Fig. 2A and B). The postoperative course was uneventful, with complete resolution of abdominal pain and vomiting. Fetal viability was confirmed postoperatively, and the patient was discharged home in stable condition. The patient was followed by the obstetric and surgical teams with no noted complications.

## Discussion

### Clinical characteristics

Internal herniation through a defect of the broad ligament of the uterus is an uncommon cause of small-bowel obstruction (SBO) and represents a small subset of internal hernias. Clinically, The reported patients are typically women of reproductive age, most frequently between the third and fifth decades of life, with possible associated congenital or acquired risk factors (e.g. prior pelvic surgery, cesarean delivery, multiparity, obstetric trauma, or inflammatory processes). Nevertheless, several cases occur without identifiable risk factors, supporting a congenital origin in a proportion of patients. Major systemic comorbidities (e.g. Diabetes Mellitus) are variably reported and are not considered defining features of the disease spectrum [1–4]. In pregnancy, diagnostic interpretation becomes more complex, as non-specficic symptomology (e.g. abdominal pain, nausea, vomiting) may overlap with common gestational physiology or benign obstetric conditions, predisposing clinicians to anchoring bias and potentially delaying recognition of a mechanical obstruction [5].

### Diagnostic process

Broad-ligament hernias frequently manifest with nonspecific or intermittent symptoms, while classical peritoneal signs may be absent or late. Across the literature, the most commonly described features resemble those of SBO, including colicky abdominal pain, vomiting, distension, and obstipation, although intensity and progression vary considerably. Furthermore, despite advances in radiology, many diagnoses remain intra-operative. Imaging may demonstrate dilated or abnormally positioned small-bowel loops adjacent to the uterus, yet clear visualization of the ligamentous defect is uncommon. Several published reports emphasize that definitive identification is frequently achieved only during surgical exploration. Our patient followed a similar trajectory, in which persistent symptoms and laboratory evolution prompted operative confirmation after initially equivocal assessment [1–4].

Regarding imaging safety in early gestation, there is no absolute prohibition. The American College of Obstetricians and Gynecologists recommends ultrasonography and MRI when adequate, but emphasizes that CT should not be withheld if necessary for maternal care, because most diagnostic exposures are below levels associated with fetal harm [5]. Likewise, the American College of Radiology advocates a benefit–risk approach, noting that routine studies typically deliver uterine doses far beneath deterministic thresholds, while advising additional caution for higher-dose pelvic examinations. Therefore, imaging choice should be directed by the clinical question and urgency rather than fear of radiation-induced teratogenicity [5, 6].

### Management and prognosis

The initial conservative therapy for suspected SBO in pregnancy includes bowel rest, intravenous fluids, electrolyte optimization, antiemetics or analgesia, and nasogastric decompression when indicated (e.g. frequent vomitus), accompanied by close reassessment and early multidisciplinary input. This strategy is acceptable only in the absence of suspected strangulation and under vigilant monitoring [1, 4]. The failure of conservative management is suggested by persistent or worsening pain, continued vomiting, inability to tolerate intake, deteriorating laboratory parameters, or imaging findings concerning for unresolved obstruction (e.g. dilation of bowel, air-fluid levels), as with internal hernias, prolonged observation increases the likelihood of progression to a closed-loop configuration and possible strangulation. Therefore, indications for urgent surgery include suspected strangulation, peritonitis, hemodynamic instability, or non-resolution despite resuscitative measures, as delay exposes both mother and fetus to escalating risks of sepsis-induced complications (e.g. septic shock, abortions), which are associated with substantially greater morbidity than operative intervention itself. Accordingly, contemporary guidelines support early exploration once deterioration is evident [1–4].

The minimally invasive nature of laparoscopy has an expanding role in both diagnostics and therapeutic approaches, as multiple recent publications document successful laparoscopic reduction and defect management, with low rates of open conversion (i.e. reserved for cases requiring bowel resection or when visualization is inadequate). Importantly, society guidelines affirm that laparoscopy can be performed during pregnancy, including the first trimester, provided appropriate precautions are observed (e.g. intra-operative materno-fetal monitoring) [3, 4, 7]. In the present case, escalation to diagnostic laparoscopy after persistent symptoms and worsening inflammatory markers enabled reduction of the herniated bowel before vascular compromise developed. This experience reinforces the recurring message in the literature that timely surgical evaluation remains the most important modifiable determinant of outcome [1–4, 7].

## Conclusion

Persistent abdominal pain and vomiting in early pregnancy should not fall under anchoring diagnostic bias (i.e. be attributed solely to hyperemesis gravidarum). When symptoms fail to improve or laboratory abnormalities progress, reassessment and surgical consultation for non-obstetric causes (e.g. incarcerated hernia) are essential. Timely diagnostic laparoscopy remains a safe and effective strategy to prevent catastrophic delay and optimize maternal–fetal outcomes.
